# Mitral valve surgery after failed transcatheter edge-to-edge repair: Operative techniques and institutional experience

**DOI:** 10.1016/j.xjtc.2023.11.017

**Published:** 2023-12-10

**Authors:** Jad Malas, Rishab Humar, Qiudong Chen, Achille Peiris, Dominick Megna, Michael E. Bowdish, Joanna Chikwe, Alfredo Trento, Dominic Emerson

**Affiliations:** Department of Cardiac Surgery, Smidt Heart Institute, Cedars-Sinai Medical Center, Los Angeles, Calif

**Figure:**
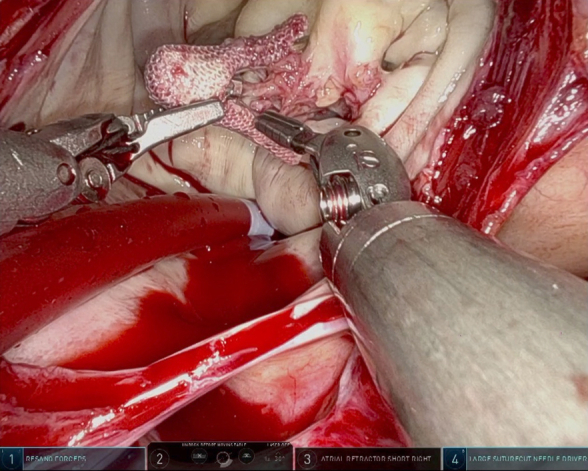
Robotic-assisted mitral repair of failed transcatheter edge-to-edge repair.

**Central Message**

Mitral surgery after failed transcatheter edge-to-edge repair poses an increasingly common challenge regarding patient selection and surgical techniques required for successful and durable repair.

Recurrent or residual mitral regurgitation (MR) after transcatheter edge-to-edge repair (TEER) poses an increasingly common challenge, with national surgical repair rates <5%.^1^, ^2^, ^3^ We report our institutional experience of mitral surgery following failed TEER and highlight 2 cases of successful repair (Video 1). This study was approved by the Institutional Review Board at Cedars-Sinai Medical Center, with a waiver of informed consent (protocol ID: STUDY00001188; approval date February 19, 2021).

## Case 1

A 38-year-old man presented with cardiogenic shock. Workup revealed severe MR due to P2 prolapse and an ejection fraction of 25%. After multidisciplinary discussion, temporizing TEER was performed. Three clips were placed—the degree of MR was reduced from severe to mild. However, 4 months after TEER he developed recurrent severe MR, and a robotic mitral repair was performed. All 3 clips were removed with preservation of native leaflets. A standard P2 triangular resection was performed and a 42-mm true-sized flexible annuloplasty band was placed. Postbypass transesophageal echocardiogram revealed trace MR and excellent biventricular function. The patient was discharged on postoperative day 5 after an uneventful hospital course.

## Case 2

A 60-year-old man with severe MR due to P2 prolapse developed dyspnea on exertion. Despite extensive counseling, he refused surgery and opted for TEER; 3 clips were placed with a reduction in MR from severe to mild-moderate. The patient remained asymptomatic for 2 years; however, he developed recurrent dyspnea with severe MR and robotic mitral repair was performed. Three clips were removed, with considerable raw surface of both anterior and posterior leaflets necessitating extensive debridement. Subsequently, a triangular resection of P2 was performed. A secondary chord from P2 was transposed to A3. A saline test was performed demonstrating residual MR necessitating additional repair. Edge-to-edge repair of A3 to P3 and A2 to P2 was performed, and a 38-mm true-sized flexible annuloplasty band was placed. Post-bypass transesophageal echocardiogram revealed mild residual MR with a mean transmitral gradient of 4 mm Hg. The patient was discharged on postoperative day 4 after an uneventful hospital course.

## Institutional Experience

Between 2011 and 2023, we have performed first-time surgical mitral intervention in 48 patients with failed TEER. Repair was performed in 19% (9 out of 48), including 28% (9 out of 32) with degenerative disease. Among those undergoing repair, 100% remained alive and 89% had freedom from 2+ mitral regurgitation at a median follow-up of 2 years.

## Conclusions

Mitral surgery after TEER is an increasingly common surgical challenge, and our institution has been able to perform successful repair at a rate considerably higher than the national average.

## Conflict of Interest Statement

Drs Malas and Chen are supported by grants from the National Institutes of Health for Advanced Heart Disease Research (T32HL116273). Dr Chikwe serves as the primary investigator on the Percutaneous or Surgical Mitral Valve Repair Trial (NCT05051033). All other authors reported no conflicts of interest.

The *Journal* policy requires editors and reviewers to disclose conflicts of interest and to decline handling manuscripts for which they may have a conflict of interest. The editors and reviewers of this article have no conflicts of interest.

## Supplementary Data

Video 1 Robotic assisted mitral valve repair following failed transcatheter edge-to-edge repair. Video available at: https://www.jtcvs.org/article/S2666-2507(23)00469-8/fulltext.

## References

[1] Chikwe J., O'Gara P., Fremes S., Sundt T.M., Habib R.H., Gammie J. (2021). Mitral surgery after transcatheter edge-to-edge repair: Society of Thoracic Surgeons Database analysis. J Am Coll Cardiol.

[2] Kaneko T., Hirji S., Zaid S., Lange R., Kempfert J., Conradi L. (2021). Mitral valve surgery after transcatheter edge-to-edge repair: mid-term outcomes from the CUTTING-EDGE International Registry. JACC Cardiovasc Interv.

[3] Young M.N., Kearing S., Albaghdadi M.A., Latib A., Iribarne A. (2022). Trends in transcatheter versus surgical mitral valve repair among medicare beneficiaries, 2012 to 2019. JAMA Cardiol.

