# Supplementary figures and images for: Mitral valve surgery after failed transcatheter edge-to-edge repair: Operative techniques and institutional experience
Source: JTCVS Tech. 2023 Dec 10;23:47–8. doi: 10.1016/j.xjtc.2023.11.017 (PMC10859654; doi:10.1016/j.xjtc.2023.11.017)

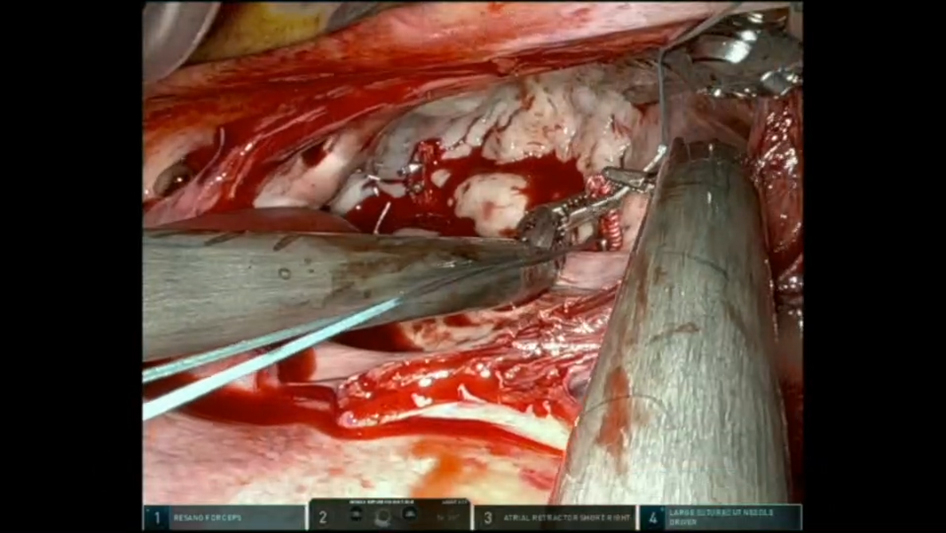

Supplement: Video 1 — Robotic assisted mitral valve repair following failed transcatheter edge-to-edge repair. Video available at: https://www.jtcvs.org/article/S2666-2507(23)00469-8/fulltext. [file fx2.jpg]
